# Unscrambling light—automatically undoing strong mixing between modes

**DOI:** 10.1038/lsa.2017.110

**Published:** 2017-12-01

**Authors:** Andrea Annoni, Emanuele Guglielmi, Marco Carminati, Giorgio Ferrari, Marco Sampietro, David AB Miller, Andrea Melloni, Francesco Morichetti

**Affiliations:** 1 Dipartimento di Elettronica, Informazione e Bioingegneria, Politecnico di Milano, Milano 20133, Italy; 2 Ginzton Laboratory, Stanford University, Spilker Building, Stanford, CA 94305, USA

**Keywords:** optical processing, photonic integrated circuits, silicon photonics, tuneable photonic devices

## Abstract

Propagation of light beams through scattering or multimode systems may lead to the randomization of the spatial coherence of the light. Although information is not lost, its recovery requires a coherent interferometric reconstruction of the original signals, which have been scrambled into the modes of the scattering system. Here we show that we can automatically unscramble optical beams that have been arbitrarily mixed in a multimode waveguide, undoing the scattering and mixing between the spatial modes through a mesh of silicon photonics tuneable beam splitters. Transparent light detectors integrated in a photonic chip are used to directly monitor the evolution of each mode along the mesh, allowing sequential tuning and adaptive individual feedback control of each beam splitter. The entire mesh self-configures automatically through a progressive tuning algorithm and resets itself after significantly perturbing the mixing, without turning off the beams. We demonstrate information recovery by the simultaneous unscrambling, sorting and tracking of four mixed modes, with residual cross-talk of −20 dB between the beams. Circuit partitioning assisted by transparent detectors enables scalability to meshes with a higher port count and to a higher number of modes without a proportionate increase in the control complexity. The principle of self-configuring and self-resetting in optical systems should be applicable in a wide range of optical applications.

## Introduction

When a coherent light beam passes through an optical object, interference from scattering or different paths can distort the beam. Strong diffuse scatterers^1, 2^ and even simple multimode fibres or waveguides^3, 4^ can generate complex speckle patterns from simple beams, giving rise to strong scrambling of multiple beams and scrambling any information on these beams^5^. Historically, for beams of the same wavelength and polarization, an efficient approach for the separation of these beams and channels optically has not been available. This problem is worse if the characteristics of the optical object are not known, and becomes even more severe if the object changes over time.

If the object is measured interferometrically or if some global feedback algorithm is used, the input field required for a desired output field can be calculated^1, 2, 4^. A spatial light modulator can set up any one such input field from an input beam, but it cannot simultaneously construct multiple arbitrary overlapping input fields from multiple inputs. In few mode optical fibres or waveguides, specific modes can be separated based on their symmetries and/or different phase velocities^6, 7, 8, 9, 10^, and signals in well-defined modes can be interchanged or switched^11, 12^. However, for arbitrary orthogonal input beams and/or for beams that couple or scatter during the propagation due to imperfections or bends, such approaches cannot generally separate the resulting complex superpositions of output guided modes. Though information can be recovered by coherent detection together with analogue-to-digital conversion and digital electronic multiple input multiple output (MIMO) processing^13, 14^, these approaches require complex digital circuits with associated power, speed and capacity limits.

**Figure 1:**
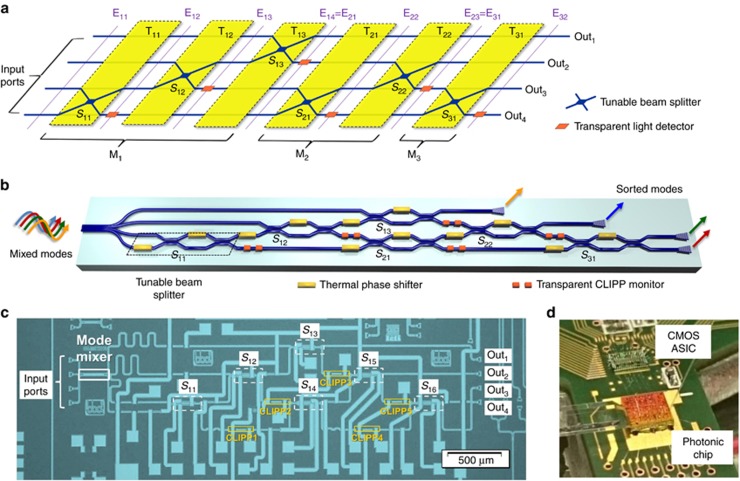
Self-configuring mode unscrambler integrated in a silicon photonic chip. (**a**) Schematic concept of an *N* × *N* (*N*=4) triangular mesh of tuneable beam splitters implementing any arbitrary transformation on *N*-dimensional input vectors. Transparent detectors at the output port of each beam splitter monitor the evolution of the optical field **E**_*m,k*_ along the entire mesh enabling local control operation on each beam splitter individually. (**b**) Guided-wave implementation of the mesh through a lattice of two-port cascaded MZIs realizing the tuneable beam splitters controlled through a pair of integrated phase shifters. (**c**) Silicon photonic four-mode unscrambler consisting of six thermally actuated MZIs individually monitored by transparent CLIPP detectors. Mode scrambling is induced on chip through a multimode waveguide section (mode mixer). Self-configuration and stabilization of the circuit is performed through a CMOS ASIC (**d**) bridged to the silicon chip, which is connected to an FPGA controller.

In waveguides where the loss is essentially the same in all the different propagating modes, in principle, the scattering between the modes can be undone with a unitary linear processor (or, in practice, a processor with uniform loss across all channels). A triangular mesh of 2 × 2 tuneable beam splitters (Figure 1a) is a well-known architecture for the implementation of arbitrary unitary operations^15^ (see Supplementary Note 1 for discussion of other meshes). While such meshes were successfully implemented in integrated photonics for quantum applications^16, 17, 18^, progressive self-configuring algorithms^19, 20, 21, 22^ were not yet available, leading to the use of time-consuming global calibration and optimization algorithms. Self-configuration of a triangular mesh has been recently demonstrated, though that demonstration was limited to automatic coupling of one input beam and to the rerouting of a single signal through an optical switching matrix^23^.

Here we demonstrate that strong mixing between modes can be undone all-optically, automatically and without any advance knowledge of the mixing object’s details; furthermore, our approach can adapt to changes in the mixing object in real time. We explicitly demonstrate the separation and reconstruction of four optical beams after these beams were completely and arbitrarily mixed in a multimode waveguide. We use a silicon photonic mesh architecture^15^ with built-in transparent contactless integrated photonic probe (CLIPP) detectors^24^ and progressive self-configuring algorithms^19, 20, 21, 22^ to demonstrate simultaneous unscrambling, sorting and tracking of the four beams, and information recovery from four mixed mode-division-multiplexed (MDM) channels. We also then strongly perturb the guide so that the channels are completely mixed again, and demonstrate the automatic recovery of the mode separation.

## Materials and methods

### Self-configuration of the mesh

An *N × N* triangular array or mesh of *N*(*N*-1)/2 tuneable 2 × 2 beam splitters (*S*_*m,k*_) is connected according to the mesh and photodetector topology shown in Figure 1a^19^. This mesh enables the factorization of an arbitrary *N* × *N* unitary transformation, described by the transmission matrix **H**_mesh_, into a sequence of simple 2 × 2 unitary transformations^15, 20^. The evolution of the optical field **E** along the mesh is given by^15^


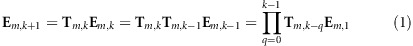


and can be described by the product of *N* × *N* transmission matrices **T**_*m,k*_ associated with each beam splitter *S*_*m,k*_, where *m*=1,2, … *N*-1 is the progressive index of the mode reconstructed at the output port Out_*m*_ and *k*=1, 2, … *N*-*m* is the *k*-th step performed to extract the *m*-th mode (see also Ref. 20 for an equivalent statement of this mathematics). In this notation, **E**_*m,k*_ is a four-element vector indicating the optical fields contained in the four waveguides of the mesh at the input of stage (*m,k*), as indicated in Figure 1a. Here we mean a vector in the linear algebra sense, which is technically in a mathematical Hilbert space rather than in a geometrical space. Inside the mesh, a given original input ‘mode’ is represented by such a vector of amplitudes in all four waveguides, and, as such, it could also be referred to as a ‘supermode’ of the four guides. Each matrix **T**_*m,k*_ is an *N* × *N* matrix (4 × 4 in Figure 1a) constructed by starting with an identity matrix and then replacing the elements *T*_*ij*_ (*i, j*=*N-k, N-k+1*) with the elements of the 2 × 2 matrix **T**_*BS*_ of the tuneable beam splitter *S*_*m,k*_, given, for example, by


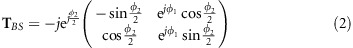


where *ϕ*_1_ and *ϕ*_2_ are controllable phase shifts (in Equation (2), subscripts *m,k* are omitted for notational simplicity). Each beam splitter *S*_*m,k*_ modifies only the components *N-k* and *N-k+1* of the field vector **E**_*m,k*_, leaving the other *N-*2 components unchanged. Importantly, at each stage of the mesh, a single transparent light detector (placed on either the waveguide *N-k* or *N-k+1*) enables us to follow the (super)mode evolution throughout the entire mesh without impairing the operation of the mesh itself.

To illustrate the self-configuration procedure, consider a first beam shining on the mesh inputs, and hence generating a vector **E**_11_ of coherent input beam amplitudes at the input ports. To output all of this beam power at port Out_1_, the beam splitters *S*_11_, *S*_12_ and *S*_13_ are progressively adjusted to cancel out the power at the embedded detectors^19, 20, 21^. Independent of the relative amplitudes and phases in the input ports, all power from the input vector **E**_11_ is automatically combined into one output beam. Mathematically, we can consider this to be a progressive multiplication^20^ by matrices **T**_11_, **T**_12_ and **T**_13_ that generates vectors **E**_12_, **E**_13_ and **E**_14,_ constructing an overall matrix **M**_1_, and progressively combining these amplitudes into the first element of **E**_14_. Since unitary operators preserve orthogonality, if we now shine a second beam with an orthogonal input vector of amplitudes into the mesh, none of that beam appears in port Out_1_. Hence, all of the second beam will pass through the transparent photodetectors into beamsplitters *S*_21_ and *S*_22_, giving the vector of amplitudes **E**_21_ in the lower three guides; those beamsplitters can then be similarly automatically aligned to couple all of the second beam to port Out_2_, implementing an additional matrix **M**_2_. Configuring each *m*-th diagonal row of the mesh, which is associated with the mode reconstruction matrix


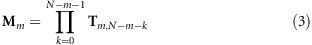


we can separate any arbitrary set of four orthogonal input vectors to the four output ports Out_1_, Out_2_, Out_3_ and Out_4_, formally implementing an arbitrary unitary transform^20^. Note that such training does not require any calibration of the phase shifters inside the mesh, and can be completed automatically and progressively in one algorithmic pass through the set of beamsplitters.

Here we described turning on the training beams one by one, with the second beam specifically not turned on until the row of beamsplitters *S*_11_, *S*_12_ and *S*_13_ is fully configured; similar procedures were conducted for further beams and beamsplitter rows. Indeed, such a separated training may always be required when working with simple continuous beams and for related progressive algorithms that can run based on detectors only at the output ports^19, 22^. If, however, the beams of interest are not mutually coherent, and if we can put an identifying ‘key’ on each such training beam, such as a small modulation at a different frequency or ‘tone’ for each beam, then the entire configuration process can be run simultaneously with all beams on at once^19^.

### Design and technology of the silicon photonic mode unscrambler

To demonstrate on-chip mode unscrambling, a silicon-photonic 4 × 4 triangular array of six Mach-Zehnder Interferometers (MZIs) was fabricated on a 220-nm silicon-on-insulator platform through a LETI-ePIXfab multi-project-wafer run (Figure 1b and 1c)^25^. Tuneable beam splitters are realized using thermally actuated balanced MZIs, with 40-μm long directional couplers (300 nm waveguide spacing in the coupling region) and 120-μm long arms spaced by 20 μm. Titanium nitride integrated heaters, with width and length of 1 and 100 μm, respectively, are used to control the phase shifts *ϕ*_1_ and *ϕ*_2_ of the MZIs, providing *π*-phase-shift with an electrical power consumption of ∼10 mW and a response time of less than 10 μs.

The optical power is locally monitored on chip by transparent CLIPP detectors integrated at the lower output port of each MZI (see Supplementary Note 2 and Supplementary Fig. S1). In the CLIPP detector^24^, surface-state absorption^26, 27, 28^ in the silicon waveguide gives photoconduction that can be detected capacitively, giving a sub-bandgap all-silicon photodetector for high-sensitivity measurement of light power in the waveguide without introducing any appreciable loss, reflection or phase perturbation in the optical field. The CLIPPs detectors consist of two 20 μm × 50 μm electrodes that are mutually spaced by 100 μm and are fabricated using the same metal layer as that used for the heaters and that is placed above the optical waveguides at the distance of ~600 nm from the Si core. At a wavelength of 1550 nm, the expected metal loss is on the order of 0.1 dB cm^−1^; this is more than one order of magnitude below the waveguide propagation loss (1.7 dB cm^−1^) and results in a negligible loss of 0.001 dB expected for the CLIPP^24^. The absence of any relevant absorption implies that the CLIPP monitors the power without introducing any differential absorption in the mesh paths, thus preserving the (super)mode orthogonality. Gold metal strips connect the thermal actuators and the CLIPPs to the 100 μm × 100 μm contact pads, where wire-bonding of the photonic chip to the external electronic circuit is performed. Experimentally, we were not able to observe any significant difference between the loss of a waveguide integrating up to 10 CLIPPs with respect to a reference waveguide (with no CLIPPs) with the same length.

Four spatially decoupled input beams (modes A, B, C and D) are injected into the silicon chip through an array of four single mode fibres, which are vertically coupled to the silicon waveguides through a commercial four-channel glass transposer (Figure 1d). The four modes are coupled to the photonic chip through two arrays of input and output grating couplers that are mutually spaced by 127 μm. Mode scrambling is intentionally induced on chip with an integrated mode mixer consisting of a straight multimode waveguide section with four single mode input/output waveguides. The design of the mode mixer was optimized to reduce the loss (see Supplementary Note 3) because loss would impair the orthogonality of the modes, thus affecting the mode reconstruction performed by the MZI mesh. The multimode waveguide is 180-μm long and 10-μm wide, and the single-mode input/output waveguides are linearly tapered up to a width of 2 μm.

**Figure 2:**
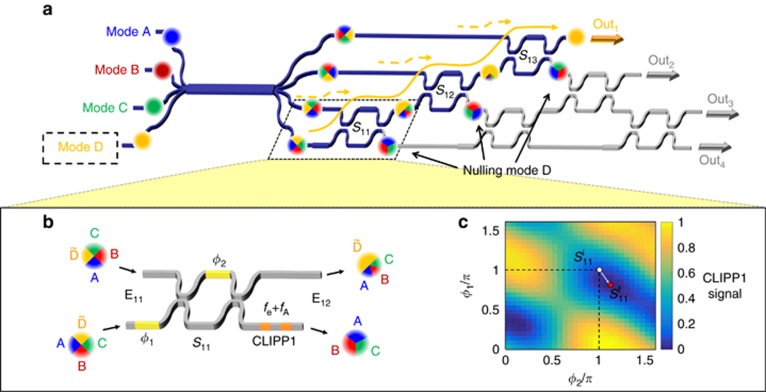
On-chip unscrambling of optical modes. (**a**) Mixed modes are reconstructed at the output port of the 4 × 4 silicon photonic mesh by sequentially tuning the MZI beam splitters. To reconstruct the first mode (*m*=1, Mode D) at port Out_1_, the first row of the mesh (**M**_1_) is configured by progressively nulling the light intensity at the lower output arms of MZI *S*_11_, *S*_12_ and *S*_13_, where a CLIPP detector is integrated (**b**). (**c**) Normalized power of Mode D measured by CLIPP1 integrated after *S*_11_. Depending on the initial MZI biasing (*S*^i^_11_), convergence to different equivalent solutions *S*^f^_11_ (local minima of the map) may be achieved.

This mode mixer can be represented by a matrix **H** that maps any vector of each input in modes A, B, C or D in Figure 2a to a resulting vector of field amplitudes at the single-mode waveguide outputs that form the inputs to the mesh. To verify the strong mixing generated by the multimode waveguide, we separately checked an identical stand-alone mode mixer (fabricated onto the same chip), showing that at a wavelength of 1525 nm, each input mode is scrambled at the output ports with ~25% power distribution (Supplementary Fig. S2) and with an excess insertion loss lower than 0.7 dB. The integrated mixer provides almost constant mode power coupling across a wavelength range of several tens of nanometres, with negligible differential group delay among the different modes. This situation emulates the mode scrambling that may occur in short links of a few-mode fibre^29^ (see ‘Results and discussion: On-chip MDM channel unscrambling’ section).

The on-chip attenuation of the MZI mesh (excluding the mode mixer) is less than 1 dB, with ~0.5 dB arising from the silicon waveguide propagation loss and the remaining 0.5 dB due to the excess insertion loss of the directional couplers used in the tuneable beam splitters. To avoid on-chip differential losses as well as to balance all interferometric paths of the mesh, folded waveguide sections are added between the different stages of the mesh. All bends throughout the circuits have a curvature radius of 20 μm, allowing very low reflections and negligible bending losses. The circuit footprint, including metal routing and contact pads, is 3.7 mm × 1.4 mm.

### Electronic platform for tuning and locking

For simultaneous read-out of all the CLIPPs integrated in the photonic circuit, a custom-designed multi-channel CMOS ASIC realized in a 0.35-μm AMS CMOS process was bridged to the silicon photonic chip and mounted on the same printed circuit board (Figure 1d)^30, 31^. The ASIC contains a low-noise front-end amplifier followed by a fully integrated lock-in system for the extraction of the in-phase and quadrature components of the light-dependent waveguide impedance^24^. The ASIC has four parallel read-out channels, with each channel featuring an 8 × input multiplexer to address up to a total of 32 CLIPPs.

When the input modes A, B, C and D are simultaneously coupled into the chip, the CLIPP detectors can identify the power associated with each mode regardless of the presence of other concurrent modes injected at other input ports and scrambled by the mode mixer. To enable mode discrimination, each mode is labelled with a weak ‘key’ or pilot tone before being coupled to the silicon chip. In previous studies, we have demonstrated that such a labelling operation can be performed without affecting the quality of the signals^32, 33^.

Sinusoidal tones with 5% peak-to-peak relative intensity are generated through external MZI lithium niobate modulators biased at the linear working point (3 dB attenuation). The tone frequency *f*_q_={4 kHz, 7 kHz, 10 kHz, 11 kHz} of the *q*-th mode (*q*=A, B, C, D) was suitably chosen to avoid mutual overlap of the overtones that can be generated by the non-perfectly linear response of the modulators. Different tone waves (for example, square waves) as well as different biasing points of the modulator could also be used to reduce the loss associated with tone generation, but such tones would require a more careful selection of the tone frequencies in order to avoid mutual overlaps. To identify the *q*-th mode, the CLIPPs are demodulated twice, first at the read-out frequency *f*_e_ around which the CLIPP sensitivity to optical power variations is maximized (~100 kHz in the reported experiments, see Supplementary Note 1), and then at the frequency *f*_*q*_, of the mode to be monitored (see Supplementary Note 4 and Supplementary Fig. S3). Second demodulation at a frequency different from *f*_*q*_ produces a very low crosstalk signal (lower than −50 dB), which is mainly due to the noise level of the electronic front-end^31, 33^.

The four output signals from the ASIC are acquired and conditioned by a customized field programmable gate array (FPGA)-based electronic platform and are digitally demodulated at the frequencies *f*_*q*_ and processed by tuneable infinite impulse response filters (down to 4 Hz bandwidth) to identify the power level of each mode. The FPGA drives the 12 heaters of the silicon photonic chip to tune and lock the 6 MZIs to the desired working points. In the experiments, the system was set to perform the CLIPP read-out in 50 ms, allowing an automatic 2D scan of each MZI (30 × 30 pixel map, as in Figure 2c) in ~45 s and automated full reconfiguration of the mesh (starting from unbiased MZIs) in ~15 s. Once the mesh is configured, tracking of time-varying mixed modes can be performed on a time scale of a few hundred milliseconds. By following the design rules and electronic read-out optimization strategies provided in other specific contributions^31, 34^, the CLIPP read-out time can be reduced by two orders of magnitude, while maintaining a sensitivity better than −20 dBm, thus enabling the tracking of mode mixing variations occurring within a millisecond range.

### Experimental set-up for on-chip unscrambling of MDM channels

**Figure 3:**
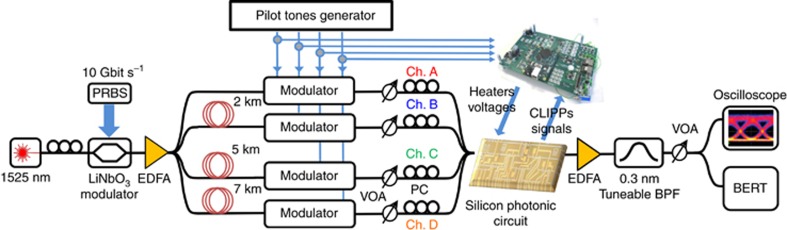
Experimental setup used for the demonstration of all-optical unscrambling of mixed MDM channels.

A detailed schematic of the experimental set-up employed for the demonstration of on-chip unscrambling of MDM channels is shown in Figure 3. The four channels encoded on modes {A, B, C, D} are generated by using a common laser source with an emission wavelength of 1525 nm that is intensity modulated at a data rate of 10 Gbit s^−1^, according to a 2^31^—1 on-off keying pseudo random bit sequence (PRBS) using a commercial LiNbO_3_ Mach-Zehnder modulator. After amplification through an erbium-doped fibre amplifier (EDFA), the modulated signal is divided by a 1 × 4 fibre optic splitter. The four data streams are de-correlated using coils of standard single-mode fibres of different lengths, introducing relative delays (>10 μs) that are much greater than the signal coherence length. Variable optical attenuators (VOAs) are employed to equalize the channel optical power to 0 dBm at the input of the silicon photonic circuit. Polarization controllers (PCs) enable the selection of the transverse electric (TE) polarization at the output of the glass transposer (see Figure 1d) in order to optimize the coupling efficiency of each channel with the optical waveguides. At the output ports of the circuit, the transmitted signals are amplified by an EDFA followed by a filter (0.3 nm bandwidth) that is added to reduce the off-band amplified spontaneous emission noise. A VOA is used to control the received power at the input of the photodetector in order to perform the BER and eye diagram measurements.

### Light-induced perturbation of the mode mixer

To modify the scrambling process responsible for the mode mixing, the integrated mode mixer was exposed to an intense light beam generated by a fibre-coupled laser source, emitting light at 980 nm with a maximum power *P*=20 dBm. A lensed fibre with a spot area *A* of ~3 μm^2^ at the focal distance is used to irradiate the mode mixer from the top with an intensity of ~3.3 MW cm^−2^. Because the spot size *A* is much smaller than the mode-mixer size (see Supplementary Note 2), only a small portion of the device is directly exposed to the light beam. To estimate the density of free carriers *N* generated in the device, we assume uniform absorption of the light across the core layer. Neglecting the reflection at the air/silica/silicon interfaces, the carrier density is governed by the following rate Equation


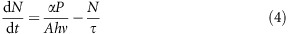


where α is the silicon absorption coefficient at 980 nm (α~100 cm^−1^) and *hν* is the photon energy (1.26 eV). In silicon waveguides, the free carrier lifetime *τ* typically ranges from a fraction of a nanosecond to several tens of nanoseconds^35^. Assuming *τ*=1 ns, the steady-state carrier density 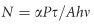 is estimated to be on the order of 2 × 10^18^ cm^−3^.

## Results and discussion

### Sorting out mixed modes

To illustrate the reconstruction of modes scrambled by propagation through the mode mixer, in the example of Figure 2a, the first row of the mesh (**M**_1_) is progressively configured to have the optical mode D reconstructed at Out_1_. In this experiment, all four input modes, which share the same optical wavelength *λ*_0_=1525 nm, are switched on, keyed by modulation tones (see Supplementary Note 4 and Supplementary Fig. S3) and injected into the silicon chip with the same power of 0 dBm.

**Figure 4:**
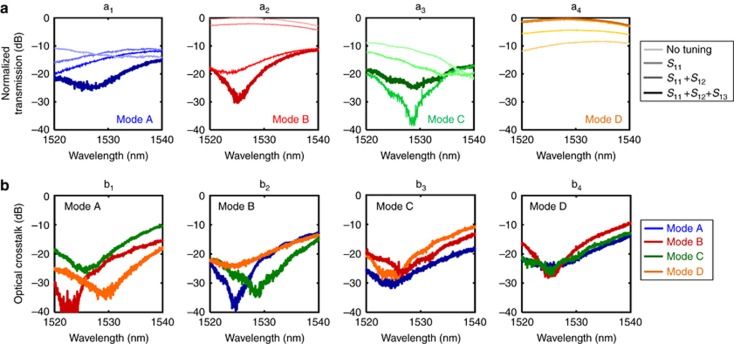
(**a**) Mesh configuration makes the transmission of the mode reconstructed at port Out_1_ progressively increase **a**_**4**_, while the crosstalk due to the concurrent modes A **a**_**1**_, B **a**_**2**_ and C **a**_**3**_ reduces. (**b**) Reconstruction of mode A **b**_**1**_, mode B **b**_**2**_, mode C **b**_**3**_, and mode D **b**_**4**_ at port Out_1_ can be achieved with less than −20 dB residual crosstalk of the three concurrent modes over a bandwidth of ~ 10 nm.

First, MZI *S*_11_ (see Figure 2b) is tuned to cancel out the power associated with mode D at the lower output port where CLIPP1 is integrated. The map presented in Figure 2c shows the intensity of mode D versus the phases (*ϕ*_1_, *ϕ*_2_) (see Figure 2b) of *S*_11_ as measured directly by CLIPP1. The thermal phase shifters are initially set to a non-zero value, such as *S*^i^_11_(*π*,*π*), in order to be able to either increase or decrease the phase shift during the tuning operation. Once convergence to a local minimum *S*^f^_11_ is achieved, the procedure is sequentially repeated through the subsequent stages *S*_12_ and *S*_13_. After the tuning of each beam splitter in the first row of the mesh (**M**_1_), the powers of the sorted mode D and of the concurrent modes A, B and C were measured at port Out_1_ over a wavelength range of 20 nm around *λ*_0_. As shown in Figure 4, although this mesh configuration process leads to a progressive increase in the output power of the reconstructed mode at Out_1_ (Figure 4a4), the crosstalk associated with each concurrent mode does not decrease monotonically as this configuration progresses (Figure 4a1-4a3). For instance, the transmitted power of mode C (Figure 4a3) reaches a minimum after the tuning of *S*_11_ and *S*_12_, yet the minimization of the overall crosstalk from all the concurrent modes results in an increased transmission of mode C after the tuning of the last stage *S*_13_. This indicates that in practice, the mesh configuration cannot necessarily be reliably achieved using only the information provided by external detectors coupled at the output ports, because convergence issues due to local minima can arise, at least if the mesh is not quite perfect. Thus, although an algorithm based only on the overall output powers may work (see the progressive algorithms using only output detectors in Ref. 19 Appendix B and in Ref. 22 and the global algorithms in Ref. 14), approaches with embedded detectors may offer faster and more robust convergence, in addition to the ability to configure the mesh when all input modes are present simultaneously.

**Figure 5:**
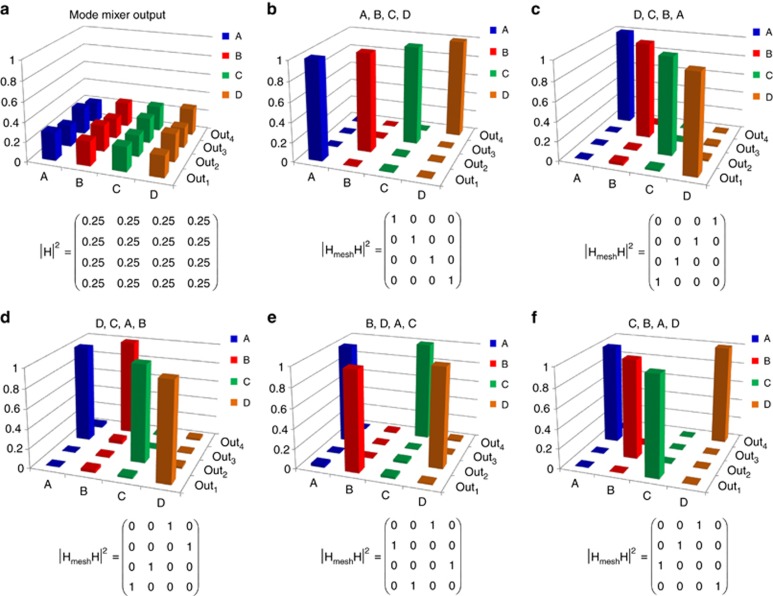
On-chip mode sorting. The mesh transmission matrix **H**_mesh_ can be configured in order to sort the reconstructed modes {A,B,C,D} arbitrarily at the output ports {Out_1_, Out_2_, Out_3_, Out_4_} of the mesh according to any 4 × 4 permutation power transmission matrix |**H**_mesh_**H**|^2^. Given the mode scrambling introduced by the mode mixer **H**, spreading the power of the input modes almost equally in the input waveguides of the mesh (**a**), panels (**b**–**f**) show the normalized light power at the output ports of the mesh, when it is configured to extract the modes in the follow order: (**b**) A, B, C, D; (**c**) D, C, B, A; (**d**) D, C, B, A; (**e**) C, A, D, B; (**f**) C, B, A, D.

Any input mode can be reconstructed at any output port with similar performance. For instance, Figure 4b shows that by properly setting **M**_1_, mode reconstruction at port Out_1_ for any particular chosen input is achieved with less than −20 dB residual crosstalk of the concurrent modes over a wavelength range of ~10 nm. More generally, the mesh transmission matrix


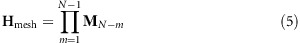


can be configured to give any desired permutation of inputs to outputs, as in a switching matrix. In other words, the overall matrix of the system |**H**_mesh_**H**|^2^ that describes the power transmission of the input modes {A, B, C, D} to the output ports of the mesh, can be chosen to take the form of a generic permutation matrix. This means that not only can the mesh perform an inversion of the **H** matrix, but the reconstructed modes can also be sorted or switched arbitrarily at the output ports. Figure 5 shows the measured light power at the output ports {Out_1_ Out_2_, Out_3_, Out_4_} for several configurations of the full mesh. In all considered cases, the power of the concurrent channels is more than 20 dB below the power of the reconstructed mode (crosstalk data are reported in Supplementary Fig. S4).

Incidentally, we note that this performance is achieved even though the intensity split ratio of the directional couplers of the MZIs is quite far from the ideal 50:50 condition (we estimate ~72% coupling in the fabricated device at 1525 nm wavelength). Numerical simulations show that an optical crosstalk lower than −25 dB is maintained up to a split ratio of ~0.75, thus implying that no significant performance degradation occurs for relative deviations as large as 50% from the ideal condition (see Supplementary Note 5 and Supplementary Fig. S5). Recent approaches may allow yet further performance optimization even with such imperfect directional couplers^22, 36^ and/or with broadband couplers^37^.

### On-chip MDM channel unscrambling

**Figure 6:**
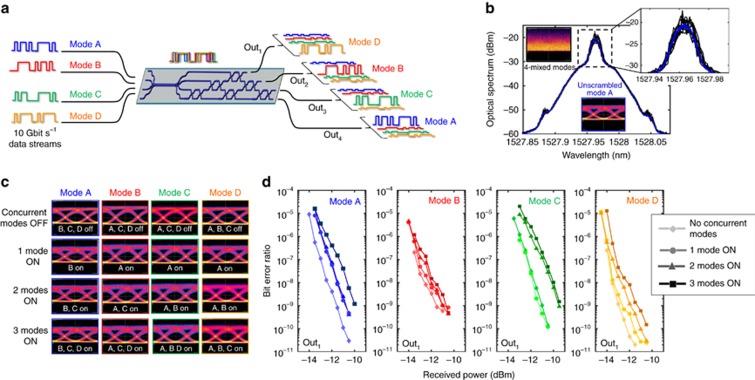
On-chip unscrambling of MDM optical channels. (**a**) Information encoded in four scrambled 10 Gbit s^−1^ intensity modulated MDM channels is recovered after mode reconstruction performed by the silicon photonic mesh. (**b**) As a consequence of mode mixing, the spectrum of the four mixed channels (black curves) exhibits deep time-varying oscillations, which disappear after mode reconstruction (mode A, blue curves). Displayed curves refer to 10 successive measurements taken at output port Out_1._ The corresponding time domain signals are shown in the eye diagrams in the insets. Eye diagram (**c**) and BER (**d**) measurements (port Out_1_) demonstrate that information encoded in each channel can be retrieved with a very small power penalty independent of the number of mixed modes.

To demonstrate the recovery of the information encoded in the optical modes undergoing the mixing process, we injected four data channels (see Figure 6a) that were all at a wavelength of 1525 nm, on separate fibres to form the inputs A, B, C and D. When the mesh is not configured, mode mixing results in deep time variations in the spectrum of the optical signal measured at the device output (Out_1_ in Figure 6b) due to the coherent beating of the four spectrally overlapped channels. In contrast, the spectrum of the reconstructed channel exhibits only a tiny frequency-domain ripple due to the residual −20 dB crosstalk of the three concurrent channels. The mode mixing leads to the complete closure of the signal eye diagram (insets of Figure 6b), which is effectively restored after mode unscrambling. The panels in Figure 6c show the eye diagrams of each reconstructed channel at port Out_1_ and do not show any deterioration as more concurrent channels are switched on. Bit error rate (BER) measurements (Figure 6d) show a power penalty <2 dB at a BER of 10^−9^ as additional channels are turned on.

**Figure 7:**
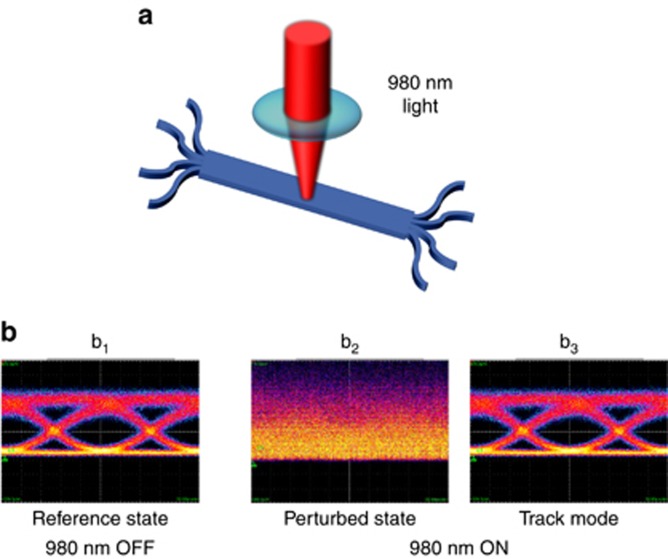
Reconstruction of modes scrambled by time-varying mixing. (**a**) A light source (980 nm) is used to perturb the mode mixer integrated in the silicon chip in order to modify the relative amplitude and phase of the mixed modes. (**b**) After configuring the mesh to reconstruct channel A at port Out_1_ (reference state, **b**_**1**_), the 980-nm source is switched on to modify the mode mixing, thus impairing mode reconstruction at the mesh output (perturbed state, **b**_**2**_). In the track mode **b**_**3**_, the mesh adaptively self-configures by controlling each MZI through a local feedback loop, in order to automatically compensate against time-varying mixing of the modes.

The MZI mesh can also self-configure automatically to track modes that are mixed by a time-varying scrambling process. The integrated mode mixer was deliberately perturbed by shining a 980-nm-wavelength light beam with an intensity of 3.3 MW cm^−2^ on it (see Figure 7a). Absorption of this light in the silicon of the mode mixer generates free carriers with a density of ~10^18^ cm^−3^, and leads to local changes in the refractive index, arising both from free carrier dispersion (blueshift) and thermal (redshift) effects^35^. These changes affect the self-imaging process along the multimode silicon waveguide^38^ and modify the mode mixer behaviour (Figure 7b). When the 980-nm beam was off (Figure 7b1), the mesh was configured to a reference state where a given channel (A) is reconstructed at one output port (Out_1_). With the 980-nm beam on, the mode mixer (Figure 7b2) is sufficiently perturbed to completely impair mode reconstruction if the mesh is not adaptively configured. Figure 7b3 shows that the eye diagram of the output channel is successfully recovered after the automatic reconfiguration of the mesh. Notably, this mode reconstruction is performed without any knowledge of the perturbation introduced in the mode mixer.

Transparency to the modulation format is one of the main advantages of performing MDM unscrambling directly in the optical domain. Therefore, we do not expect any significant performance degradation if more advanced modulation formats are employed where both the amplitude and the phase of the signals are modulated. Regarding the optical bandwidth, the realized device provides less than −20 dB residual crosstalk over a bandwidth of ~10 nm, thus posing no significant limitations to the bandwidth of the optical signals that can be manipulated. The tuning strategy itself, which is based on channel labelling with low frequency pilot tones, is inherently independent of the bandwidth and modulation format of the mixed channels, which could indeed have different bandwidths and modulation formats.

Arbitrarily mixed modes can be unscrambled by the proposed mesh, provided that no significant differential mode group delay (DMGD) is accumulated in the mixing channel. This means that the mesh can operate on channels coupled into near degenerate modes (or mode groups) of a multimode fibre propagating with the same group velocity. Because the mesh cannot compensate for an accumulated DMGD, information can be effectively recovered only when the DMGD is a small fraction of the bit time duration (<5%). This situation reflects for instance the case of intradatacentre optical connections, where the length of optical links typically ranges from few tens of metres to a maximum length of 1–2 km^39^. Low values of DMGD have been demonstrated in coupled-core fibres (3.14 ps km^−1^)^40^ and in cascaded FMFs (<1.7 ps km^−1^)^41^, enabling almost DMGD-free propagation across more than 2 km at 10 Gsym s^−1^, or 500 m at 40 Gsym s^−1^.

## Conclusion

We have demonstrated all-optical mode reconstruction, unscrambling and sorting in a silicon photonic circuit using a self-reconfiguring interferometric mesh. Because each mesh element (tuneable beam splitter) is locally monitored and feedback-controlled by transparent detectors, the progressive self-configuration of the mesh is reduced to a repeated two-degrees-of-freedom problem independently of the port-count of the mesh. This feature enables the implementation of simple, accurate and robust control of arbitrarily large meshes for the manipulation of a large number of modes^20^.

For implementation on existing silicon photonic platforms, the practical limit to the mesh scalability is neither the physical size of the mesh, nor the complexity of the tuning and locking algorithm, because meshes with more than one thousands tuneable splitters and handling several tens of modes could be realized and controlled. Power consumption of thermal actuators and propagation loss of the silicon waveguide is the main barrier to scalability to a very large number of modes (see the Supplementary Information for a quantitative analysis). This overall concept of transparent on-chip monitoring and adaptive feedback control of elementary photonic elements can be extended to arbitrary mesh topologies, such as the topologies that have been recently proposed to implement programmable photonic processors^18, 42, 43, 44, 45^.

Mode unscrambling on a photonic chip can also be exploited to improve the performance of recently proposed silicon photonics devices for the manipulation of MDM optical channels^6, 7, 8, 9, 10, 11^, where a one-by-one mapping of the modes of single-mode waveguides to predetermined modes of multimode waveguides is performed. In these examples, no mode coupling in the multimode waveguide is considered, but in reality, mode mixing could be induced by sharp bending, waveguide crossing, as well as fabrication imperfections. Mode unscramblers, such as the one proposed in this work, are thus required to mitigate these effects, which are difficult to predict at design time, and which potentially also vary in time in uncooled photonic chips because of the different temperature sensitivities of the different guided modes.

While our demonstration architecture is capable of implementing any unitary (that is, loss-less) linear function between inputs and outputs, architectural extensions allow this approach to implement non-unitary linear operations also. In applications where mode mixing must be tracked with a control system that neither reaches the end of its range (endless) nor needs to reset (resetless) to avoid communication interruptions, endless phase shifters^46^ could be integrated in the tuneable beam splitters of the mesh, though necessarily at the cost of an increasing complexity of the photonic circuit. Likewise, more complex meshes would be required to unscramble optical modes that have experienced mode-dependent loss and large DMGD. For instance, it has been shown that two unitary processors as described here can be used to undo the scattering between the modes even when the losses in the modes differ substantially from each other^45^.

The approach presented here can also be extended to other semiconductor photonics platforms, such as InP, where modes are not easily separable because of the similarity of their phase velocities^12^ and where the CLIPP operation has also been successfully demonstrated^47^. We expect that this approach will find use in mode (de)multiplexers^6, 7, 8^, multimode switches^9^ mode converters^10^, switchable mode exchangers^11^ and other programmable photonic processors^18, 42, 43, 44^ for applications in a variety of different fields, such as telecommunications, imaging, sensing, secrecy, and quantum information processing^5^.

## Author contributions

FM conceived the experiments and supervised the work. AA designed the photonic chip and performed the experiments. EG developed the firmware and performed the experiments; MC designed and tested the electronic platform; GF designed the CMOS ASIC; MS supervised the implementation of the electronic platform; AA, FM and AM analysed the data; FM, AM and DM wrote the manuscript.
