# Supplementary material for: Unscrambling light—automatically undoing strong mixing between modes
Source: Light Sci Appl. 2017 Dec 1;6(12):e17110–. doi: 10.1038/lsa.2017.110 (PMC6062024; doi:10.1038/lsa.2017.110)
Supplement: Supplementary Information [file lsa2017110x1.pdf]

Supplementary Information for
"Unscrambling light – automatically undoing strong mixing between modes"

Andrea Annoni[1], Emanuele Guglielmi[1], Marco Carminati[1], Giorgio Ferrari[1], Marco Sampietro[1], David A. B. Miller[2], Andrea Melloni[1], Francesco Morichetti[1]

[1]*Dipartimento di Elettronica, Informazione e Bioingegneria, Politecnico di Milano, via Ponzio 34/5, 20133 Milano, Italy*

[2]*Ginzton Laboratory, Stanford University, Spilker Building, 348 Via Pueblo Mall, Stanford CA 94305, USA*

Correspondence: F. Morichetti, Email: francesco.morichetti@polimi.it

Supplementary note 1: Photonic mesh architectures implementing arbitrary linear operations

At present, three architectures, made up from meshes of 2×2 interferometers, are known that can implement arbitrary unitary transforms between a vector of optical input amplitudes and a corresponding vector of output amplitudes for coherent light at a given wavelength: a "triangular mesh" architecture[1-5] (which is used in our work here), a "cascaded binary tree" architecture[3], and a "rectangular mesh" architecture[6]. Of these, both "triangular mesh" and "cascaded binary tree" architectures can be configured automatically using "training" vectors of inputs and simple progressive algorithms based on detection and simple one- or two- parameter feedback minimization processes[3,4]. In these "trainable" architectures, a given linear transform is trained using vectors that are the Hermitian adjoints of the desired rows of the corresponding matrix (as we do in this work). All three of these architectures require only a number of phase shifters that corresponds to the number of real numbers required to specify an arbitrary $N \times N$ unitary matrix, and so are optimally efficient in that sense.

For non-unitary transforms (i.e., arbitrary matrices), two architectures are known: an architecture based on the singular value decomposition (SVD) of the desired matrix[4], and one based on the use of a $2N \times 2N$ unitary matrix to implement an $N \times N$ non-unitary transform by operator dilation[2]. The SVD approach can be "trainable" and has the minimum number of required phase shifters. The SVD approach can be implemented using two unitary transforms and an additional row of modulators[4]. Each such unitary transform can be implemented using any of the above unitary architectures. If "trainable" unitary transform meshes are used, the overall non-unitary function can be trained using appropriate vectors at the inputs for one of the unitary transforms, and by shining appropriate vectors back into the output for training the other unitary transform. Hence the self-configuring approach of our work could also be applied to implement "trainable" non-unitary transformations that mathematically could also undo scattering with different loss on different modes.

Supplementary note 2: Electronic read-out of the CLIPP monitor

The working principle of the CLIPP monitor is extensively discussed in Ref. [7], where the CLIPP concept was demonstrated for the first time. For completeness' sake, in this section, we briefly recall the main features related to the CLIPP operation and electronic read-out.

The CLIPP monitors the light intensity in the waveguide by measuring the light-dependent variation of the conductance ΔG of the waveguide. Non-invasive monitoring is achieved by remotely performing an impedance measurement without electrically contacting the CLIPP electrodes with the Si core. A top-view picture of one of the CLIPPs integrated in the MZI mesh used in this work is shown in Supplementary Figure S1a. To by-pass the access capacitance provided by the insulating SiO_2 top cladding, the CLIPP electrodes are AC-coupled to the Si waveguide core. The CLIPP readout requires a low-noise transimpedance amplifier (TIA) and a lock-in detection scheme that are both integrated into the CMOS ASIC connected to the silicon chip. Details on the design of the ASIC can be found in Ref. [8], where a complete block diagram of the electronic circuit is provided.

A sinusoidal voltage V_e at frequency f_e is applied to one of the CLIPP electrodes, while the current i_e at the other electrode is collected with a synchronous electrical detection architecture. Since the CLIPP is partly made by the silicon waveguide core (resistor) and partly made by the electrode-cladding interface (capacitor), the current i_e is in general out of phase with respect to the applied voltage V_e. In order to measure the conductance of the silicon waveguide, whose variation provides information on the light intensity in the waveguide, the in-phase component (real part of the complex impedance) has to be extracted. This is done externally in the FPGA by processing the acquired in-phase and quadrature components of the overall waveguide impedance.

Supplementary Figure S1b shows the electrical signal (conductance variation ΔG) provided by a stand-alone test CLIPP fabricated on the same chip of the MZI mesh as a function of the readout frequency f_e for increasing optical power level. Maximum sensitivity to optical power variation is observed around 100 kHz. At this frequency, the responsivity curve of the CLIPP (Supplementary Figure S2c) shows a sensitivity of at least -20 dBm with a dynamic range of 30 dB. This sensitivity enables accurate monitoring of each MZI tuneable beam splitter to achieve mode reconstruction with a -20 dB residual crosstalk.

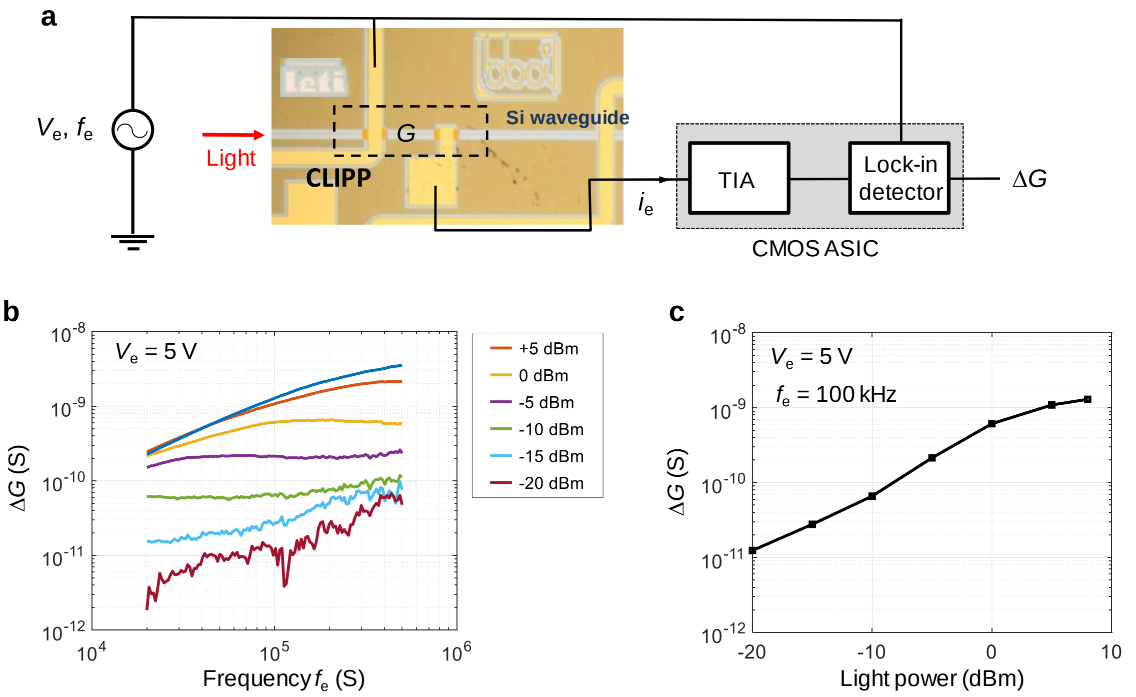

Figure S1. Performance assessment of the CLIPP monitor. (a) Top view photograph of the one of the CLIPPs integrated in the MZI mesh and block diagram of the electronic circuit integrated in the CMOS ASIC for the read–out of the CLIPP; (b) Electric signal provided by the CLIPP versus the frequency of the applied voltage signal for increasing optical power in the silicon waveguide. (c) Responsivity curve of the CLIPP measured at a frequency f_e = 100 kHz, where the sensitivity to light variation is maximum.

Supplementary note 3: Integrated mode mixer

The integrated mode mixer responsible for mode scrambling consists of a multi-mode waveguide section with four input (I_1,…, I_4) and four output (O_1,…, O_4) single mode waveguides, resulting in the multimode interference coupler shown in the schematic of Supplementary Figure S2a. Electromagnetic simulations based on the Eigenvalue Mode Expansion (EME) method were performed to optimize the design of the mode mixer in order to reduce the loss created by the imperfect self-imaging of the field at the output port of the multimode region (see Supplementary Figure S2b). To reduce the loss, the 480-nm wide single-mode input/output waveguides are linearly tapered up to a width of 2 μm. In the circuit presented in this work, the mode mixer integrated before the MZI mesh is 180 μm long and 10 μm wide. Supplementary Figure S2c shows the spectral response of a stand-alone mode mixer that was fabricated on the same chip for testing purposes. When the light is injected from one input port (due to the symmetry of the device, only curves referring to inputs I_1 and I_2 are shown) an almost-wavelength-independent 25% (± 2%) mode splitting is observed at all four output ports, thus maximizing the mode scrambling between the input modes. The overall insertion loss of the mode mixer was evaluated by comparing the sum of the power leaving the output ports to the power collected from a reference straight waveguide; for every input port an excess insertion loss lower than 0.7 dB was estimated, thus confirming that mode scrambling is performed without impairing mode orthogonality.

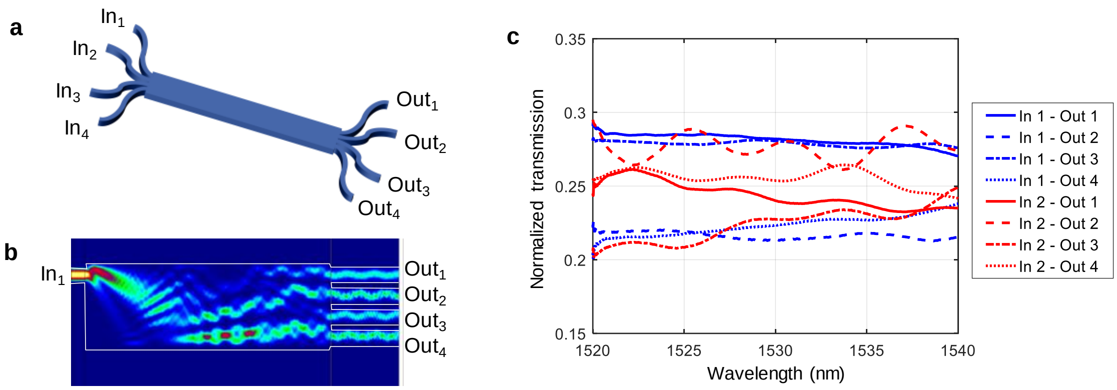

Figure S2. Optical characterization of the integrated mode mixer. (a) Schematic and (b) electromagnetic simulation of the mode mixer. (c) The fabricated mode mixer splits the input power of each input mode to all four output ports with a 25% (± 2%) split ratio over the 1520 - 1540 nm wavelength range considered in this work.

Supplementary note 4: Mode labelling and identification with modulation tones

The effectiveness of the mode identification performed by the CLIPP and its use for the monitoring of the tuneable beam splitters of the mesh is shown in Supplementary Figure S3. The three maps show the signal provided by CLIPP1 when the beam splitter S_{11} is tuned by changing the phases ϕ_1 and ϕ_2. With respect to the case where only one mode (D) is injected in the mesh (a), the presence of concurrent channels strongly modifies the map [in (b) also channel B is switched on], hindering the biasing of the MZI at the proper working point for mode D reconstruction. Mode labelling through pilot tones (c) enables monitoring and control of the state of the MZI with no side effects associated with the presence of the concurrent channels.

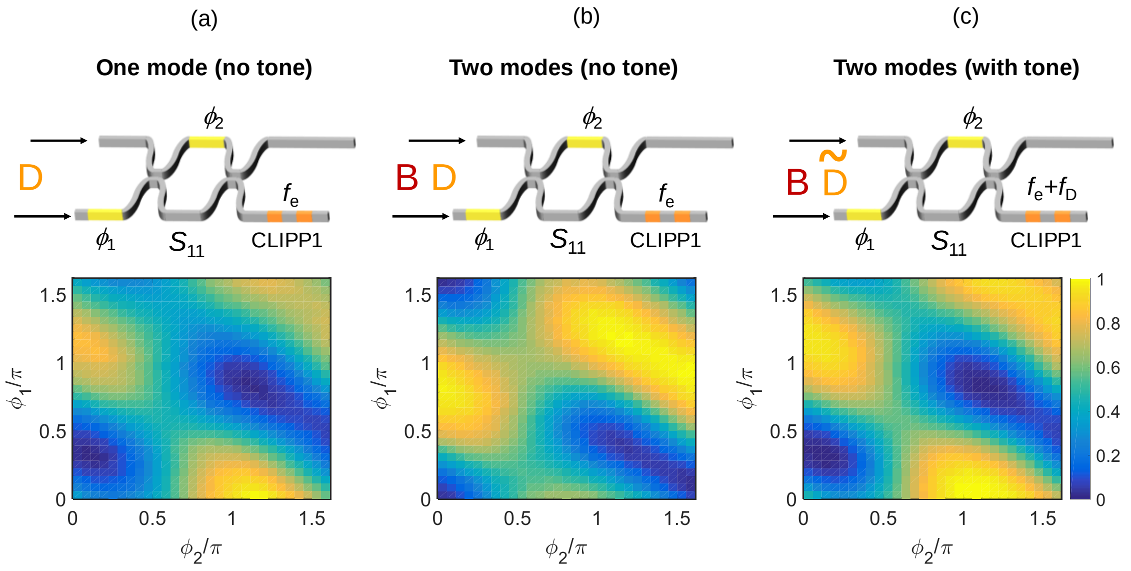

Figure S3. CLIPP-assisted monitoring of the tuneable beam splitters of the mesh by using mode labelling. Maps show the signal measured by CLIPP1 during the tuning operation of the beam splitter S_{11} as a function of ϕ_1 and ϕ_2, when: (a) only mode D is injected in the mesh, concurrent modes are off and no tone is applied; (b) concurrent mode B is switched on, no tone is applied and the CLIPP is read at frequency f_e; (c) concurrent mode B is switched on, a tone at frequency f_D is applied on mode D and the CLIPP is read at frequency $f_e + f_D$.

a Mode mixer output

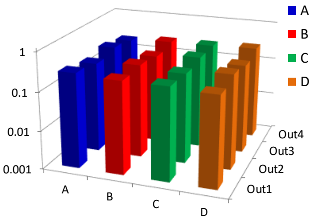

	1	2	3	4
A	-5.7	-6.6	-5.5	6.7
B	-6.0	-5.7	-6.6	-5.9
C	-6.0	-6.6	-5.9	-5.9
D	-6.6	-5.5	-6.4	5.7

b A, B, C, D

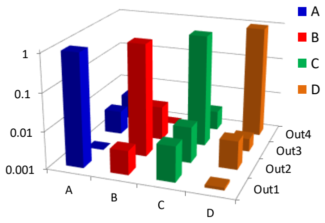

	1	2	3	4
A	-	-37.6	-23.6	-23.2
B	-23.6	-	-20.8	-34.1
C	-20.6	-20.4	-	-24.8
D	-29.3	-22.4	-23.6	-

c D, C, B, A

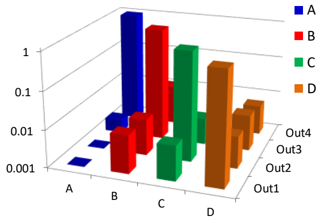

	1	2	3	4
A	-22.1	-20.5	-21.5	-
B	-27.9	-22.6	-	-20.8
C	-19.4	-	-20.4	-20.1
D	-	-26.4	-31.5	-33.0

d D, C, A, B

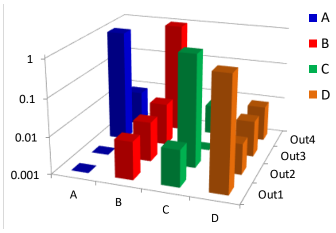

	1	2	3	4
A	-33.0	-31.5	-	-20.6
B	-20.1	-20.3	-21.0	-
C	-20.2	-	-32.7	-22.0
D	-	-21.8	-20.3	-20.5

e B, D, A, C

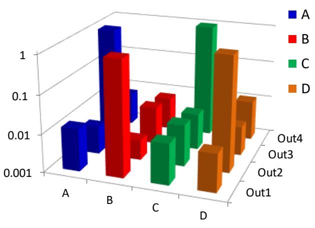

	1	2	3	4
A	-20.9	-22.8	-	-21.5
B	-	-24.8	-20.1	-21.4
C	-21.6	-20.7	-20.4	-
D	-20.1	-	-22.3	-21.2

f C, B, A, D

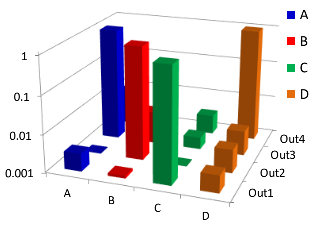

	1	2	3	4
A	-25.3	-30.3	-	-20.7
B	-29.3	-	-21.4	-31.2
C	-	-31.1	-26.8	-24.9
D	-25.4	-30.3	-23.5	-

Figure S4. Logarithm scale representation and measured crosstalk data of the permuted mode reconstructions reported in Figure 5.

Supplementary note 5: Tolerance analysis of mode reconstruction

Numerical simulations were performed by using the transmission matrix method (TMM) to investigate the sensitivity of the mesh to fabrication imperfection in the directional couplers of the MZIs. Supplementary Figure S5 shows the overall crosstalk, averaged over a bandwidth of 10 nm around 1525 nm, that is provided by the other three concurrent channels when channel A (solid blue), B (dashed red), C (dashed-dotted green), and D (dotted yellow) are respectively reconstructed at the output port Out_1. Results are reported only for split ratios > 0.5 because crosstalk curves are symmetrical with respect to the ideal condition (3 dB directional coupler). A crosstalk lower than -25 dB is observed up to a split ratio as high as 0.75 (or equivalently 0.25 for the under-coupled case), thus implying that no significant crosstalk degradation occurs for relative deviations as large as 50% from the ideal condition.

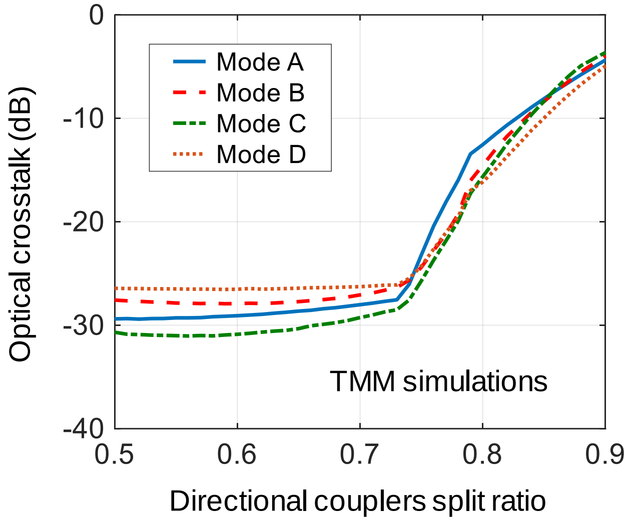

Figure S5. Robustness of mode reconstruction versus fabrication tolerances in the directional coupler of the mesh. Curves show the simulated crosstalk given by the all the concurrent channels when mode A (solid blue), B (dashed red), C (dashed-dotted green), and D (dashed yellow) is reconstructed at the port Out_1. No significant crosstalk degradation is observed up to a 50% split ratio deviation from the ideal 0.5 condition.

Supplementary note 6: Practical limits to the scalability of the mesh

In this section, we provide some information on the practical limits to the scalability of the mesh for implementation on existing silicon photonics platforms.
Given the number N of modes to be unscrambled, the number of required tunable switches (Mach-Zehnder interferometers) of the mesh scales up as $N(N\text{-}1)/2$. To give an example, unscrambling of 64 modes will require 2016 Mach-Zehnder interferometers.
In the following, we address several issues to point out where practical limits to the realization of a mesh with such a size could arise from:

Physical size of the mesh. Considering the mesh density of the fabricated device (0.25 mm^2 footprint for each Mach-Zehnder interferometer, including CLIPP monitors), the footprint of a 64 mode unscrambler would be about 5 cm^2. This size is still compatible with silicon photonics chips. However, we should consider that the mesh density of the fabricated device is not constrained by the photonic layer, but by the metal lines connecting CLIPPs and heaters to the bonding pads. The footprint of the mesh could be significantly reduced by using flip-chip technology, where the CMOS ASIC is directly bonded on top of the photonic chip, thus removing the need for most electrical wiring across the chip.

Optical loss. In the considered mesh topology, no waveguide crossings are required, so that insertion losses depend only on waveguide propagation loss and excess insertion loss in the directional couplers of the Mach-Zehnder interferometers. The maximum number of Mach-Zehnder interferometers of the mesh that are passed through by each mode increases linearly with the number of modes N. In the realized 4 mode mesh a loss of about 1 dB loss is observed; the loss increases to about 16 dB for a 64 mode unscramble realized with the same silicon photonics technology.

Electrical power dissipation. The thermal actuators employed in this work require about 10 mW for a π shift, resulting in a maximum power consumption of 120 mW for the configuration of the full mesh (integrating 12 heaters). A 64 mode unscrambler with 2016 Mach-Zehnder interferometers (4032 heaters) would thus require an unpractically high dissipation of about 40 W. Therefore, alternative phase actuators or low-power consumption heaters are required to enable scalability of the mesh to a large number of modes.

Tuning and control. One of the main benefits of the proposed progressive self-configuring algorithms is that it can work independently of the mesh size. However, since the mesh is configured through a step-by-step algorithm, the time required for the full configuration of the mesh scales up linearly with the number of mesh elements (that is quadratically with the number of mixed modes N). This issue could be overcome by using more advanced tuning algorithms. For instance, one can think to parallelize the tuning of some mesh elements that are not interferometrically connected, such as tunable splitters S_{13} and S_{21} of the mesh employed in this work. In addition, CLIPP detectors would enable partitioning of the mesh in small clusters of Mach-Zehnder interferometers, which could be locally monitored and simultaneously tuned by using multi-degree-of-freedom algorithms.

Therefore, for implementation on existing silicon photonic platforms, power consumption of thermal actuators and propagation loss of the silicon waveguide represent today the main barrier to the scalability of the mesh to a large number of modes.

References

1 Reck M, Zeilinger A, Bernstein HJ, Bertani P. Experimental realization of any discrete unitary operator. Phys Rev Let 1994; **73**: 58-61.

2 Carolan J, Harrold C, Sparrow C, Martín-López E, Russell NJ et al. Universal linear optics. Science 2015; **349**: 711-716.

3 Miller DAB. Self-aligning universal beam coupler. Opt Express 2013; 21: 6360-6370.

4 Miller DAB. Self-configuring universal linear optical component. Photonics Res 2013; **1**: 1-15.

5 Ribeiro A, Ruocco A, Vanacker L, Bogaerts W. Demonstration of a 4 × 4-port universal linear circuit. Optica 2016; **3**: 1348-1357.

6 Clements WR, Humphreys PC, Metcalf BJ, Kolthammer WS, Walmsley IA. Optimal design for universal multiport interferometers. Optica 2016; **3**: 1460-1465.

7 Morichetti F, Grillanda S, Carminati M, Ferrari G, Sampietro M et al. Non-invasive on-chip light observation by contactless waveguide conductivity monitoring. IEEE J Sel Top Quantum Electron 2014; **20**: 292-301.

8 Ciccarella P, Carminati M, Ferrari G, Bianchi D, Grillanda S et al. Impedance-sensing CMOS chip for noninvasive light detection in integrated photonics. IEEE Trans Circuit Syst II: Express Briefs 2016; **63**: 929-933.
